# Is Elevated Hba1c Level Associated with Achilles Tendon Contracture Development in Diabetic Foot Patients?

**DOI:** 10.5704/MOJ.2203.010

**Published:** 2022-03

**Authors:** RA Primadhi, HN Rasyid

**Affiliations:** Department of Orthopaedics and Traumatology, Universitas Padjadjaran, Bandung, Indonesia

**Keywords:** diabetes, ulcer, HbA1c, contracture

## Abstract

**Introduction:**

Chronic hyperglycemia can increase extracellular matrix (ECM) accumulation that leads to tendon fibrosis and subsequent contracture. Considering the reversibility of fibrosis, it is important to identify factors that are associated with it. The purpose of this study was to determine whether elevated HbA1c levels are associated with the development of Achilles tendon contracture in diabetes mellitus patients.

**Materials and methods:**

We reviewed 206 patients with diabetic foot problems between January 2015-December 2019. Demographic data, the presence of Achilles tendon contracture, and laboratory results were documented and statistically analysed between patients with contracture and without contracture.

**Results:**

Patients’ mean age was 58.46 ± 6.67 years old. Contracture was found in 84 out of 206 patients (40.78%) patients, with female predominance (45/84 patients; 53%). While contracture was found significantly associated with sex difference (0.035) and age groups (p=0.006), there was no meaningful association with HbA1c level groups (p=0.324).

**Conclusion:**

Findings do not support the use of HbA1c level as a sole determinant of Achilles tendon contracture. Seemingly, there are other confounding factors affecting the Achilles tendon contracture development in diabetes mellitus patients. It should be emphasised that albeit the association between chronic hyperglycemia and contracture development, the low HbA1c should not be overlooked as having no risk of Achilles tendon contracture and vice versa.

## Introduction

Diabetic foot pathologies are common in diabetics and pose serious health problems for developing countries. It is estimated that the annual population-based incidence of diabetic foot ulcer ranges from 1.0% to 4.1%^1^. Restricted ankle joint dorsiflexion during gait has been associated with an increased risk of diabetic foot ulceration. Other than ulceration, the development of Charcot neuroarthropathy have been linked to tightness or shortening of the Achilles tendon. In the presence of peripheral neuropathy, increased plantarflexion caused by equinus deformity can affect joint forces and gait patterns leading to joint changes. There is increasing evidence that static stiffness of the Achilles tendon may be, in part, responsible for the increased forefoot loading that might initiate the pathologic process^2-5^.

**Fig. 1:**
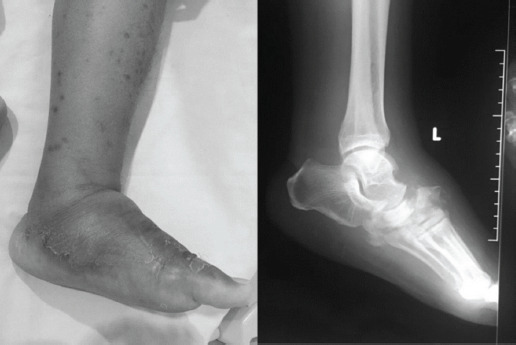
Clinical picture and radiograph of a diabetic Charcot arthropathy patient with limited ankle dorsiflexion due to Achilles tendon contracture.

One of the origins of limited ankle joint mobility (Fig 1) is Achilles tendon contracture due to fibrosis. Fibrosis is characterised by extracellular matrix accumulation (ECM) and often by a change in the quality of the ECM. It is a common pathological response to tissue insults including hyperglycemia. Experimental data had been supported causative roles for hyperglycemia and the related biochemical pathways in causing alterations in ECM turnover^6^.

Equinus, resulting from limited ankle dorsiflexion, is known to be more prevalent in diabetic patients than nondiabetic patients. There was a threefold risk of equinus in the diabetic population^3^. To the best of our knowledge, there is no prior studies on the association of diabetes chronicity and Achilles tendon contracture or fibrosis.

Glycated haemoglobin (HbA1c) reflects glycemic control over a period of time. HbA1c is a readily available laboratory test that measures the proportion of glycosylated haemoglobin in the blood and reflects the average blood glucose level over the preceding two or three months. The endocrinology literature has demonstrated that patients with higher HbA1c levels are at increased risk for the systemic complications of diabetes mellitus^7,8^. Prior studies had revealed that there was a link between HbA1c level and the

future development of tissue fibrosis, such as in cystic fibrosis and stenosing flexor tenosynovitis of the hand^8-10^.

There was evidence that mild to moderate fibrosis is reversible. In liver fibrosis patients, those who are successfully treated for HCV infection and test HCV RNA-negative showed no evidence of the fibrosis upon repeat biopsy^11^. Strict glycemic control and maintenance of normal HbA1c levels can minimise retinopathy, neuropathy, and nephropathy^8^. The similar interconnection between glycemic control and tendon fibrosis reversibility remains unclear. Considering the reversible nature of fibrosis in numerous organs, this study is conceivably to be a premise in further studies concerning the treatment strategy to reduce the tendon fibrosis in diabetic patients.

The aim of this study was to compare HbA1c result and Achilles tendon contracture in diabetic patients. Thus, this study can describe the glycemic control influence on the pathogenesis of tendon contracture. The possibility of whether HbA1c assessment could be adjunctive tools in the early diagnosis and future management of diabetic ulcer resulting from Achilles tendon contracture could be explored.

## Materials and Methods

We retrospectively reviewed the hospital database to identify diabetic foot patients, who had come to the Orthopaedic Foot and Ankle Clinics, Hasan Sadikin Hospital, a regional referral hospital in Bandung, Indonesia between January 2015 and December 2019. This study was approved by Research Ethics Committees of Hasan Sadikin Hospital before commenced.

**Fig. 2:**
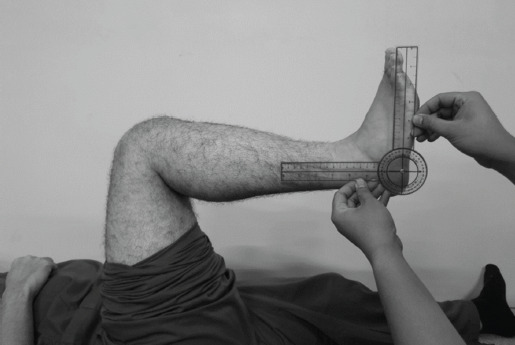
Ankle joint range of motion examination using goniometer, with 90° knee flexion.

The patients’ chart with International Classification of Disease-10 (ICD-10) codes E11.5, E11.61, and E11.621 (type 2 diabetes mellitus with circulatory complications, type 2 diabetes mellitus with diabetic arthropathy, and type 2 diabetes mellitus with foot ulcer, respectively) were reviewed in detail. We documented patients’ age, sex, presence of Achilles tendon contracture, and HbA1c levels. Achilles tendon contracture was diagnosed by measuring a limited ankle range of motion of less than 10° in knee 90° flexed using a goniometer (Fig 2)^12^. In this study we excluded patients who had other aetiology for equinus such as bony deformity, posttraumatic scarring, prolonged immobilisation, or congenital problems. Rather than analysing HbA1c as grouped data, the actual HbA1c levels were used. We used the HbA1c value closest to the date of diagnosis, not longer than three months prior to the examination. This time frame was selected because HbA1c reflects diabetic control over the preceding three months.

We performed comparison testing using Pearson chi-square analysis and Fisher exact test to determine significance. Mann Whitney test was used for analysis which the data were grouped or did not meet the chi-square requirement. Significance level was set at p=0.05. Statistical analysis was reviewed by an independent statistician.

## Results

Out of all the patients, 206 patients were included to the study. The unused patients were because of incomplete or inaccessible data. There were also duplicated patient entries due to multiple follow-up attendance. We picked their latest follow-up data to be included to this study.

We found significantly more women in the contracture group than in the non-contracture group (53% vs 45%; p=0.035). The average age in patients with contracture was 59.20 ± 5.62 years, compared with 58.61 ± 6.77 years in the noncontracture group (p=0.006). Table I shows a statistically insignificant difference in the distribution of patient by HbA1c range (p=0.324).

**Table I:** Patients characteristics and statistical analysis result

	With Contracture (N=84)		Without contracture (N=122)	p value
Sex (n [%])				
Male	39 (47%)		67 (55%)	p=0.035
Female	45 (53%)		55 (45%)	
Age (means ± SD)	59.20 ± 6.52		58.61 ± 6.77	
Age groups				
<40 years old	0		0	p=0.006
40-49 years old	2		14	
50-59 years old	44		72	
60-69 years old	32		30	
≥ 70 years old	6		6	
HbA1c (%) (median ± SD)		7.80 ± 1.67		
HbA1c	8.24 ± 1.67		7.66 ± 1.87	P=0.324

## Discussion

Previous studies have found that equinus due to Achilles tendon contracture and concomitant increased plantar forefoot pressures is more prevalent in diabetic patients. These high plantar pressures are significantly associated with elevated risks of plantar ulcerations in the presence of neuropathy^3^. Because of the potential risk that equinus deformity poses to diabetic patients, there has been increased interest in determining an association between glycemic control and the development of contracture due to fibrosis formation^3,13^.

HbA1c is now formally endorsed in many countries as a diagnostic test for type 2 diabetes as well as for monitoring. HbA1c gives an indication of chronic hyperglycemia rather than being a test of glycemia at a single point in time. It gives an integrated index of glycemia over the entire 120-day lifespan of the red blood cell. It therefore seems logical that such a test would be appropriate in diagnosing a disease characterised by chronic hyperglycemia and a gradual progression to complications. However, there are several disadvantages of using HbA1c, as for example it may vary with age and between different ethnic groups, more expensive, and being unaffordable in many low-income country situations. The results can also be affected by haemolysis and other conditions with increased red cell turnover or conditions with reduced red cell turnover e.g. iron deficiency or in any other chronic disease state^14^.

Hyperglycemia in diabetic patients is responsible for the presence of high levels of nonenzymatically produced advanced glycation end-products (AGEs). AGEs play important role in cell signaling by interacting with specific receptors that link to the activation of adhesion molecules, proinflammatory cytokines and growth factors, thus contributing to the pathogenesis of diabetic complications^6^. AGEs are able to stimulate directly the production of extracellular matrix (ECM)^15^. However, why contractures are not commonly found in all diabetic patients is not yet fully understood. Other than hyperglycemia, factors that regulate ECM formation include multiple forms of growth factors. The important role of growth factors in the pathogenesis of diabetic long-term complications was suggested by their increased concentrations in target tissues. An excess of growth factor is implicated in tissues where fibrosis predominates, whereas a lack of growth factors occurs in diabetic neuropathy and wound healing^6^. Cigarette smoking also has deleterious effects on the musculoskeletal system. In addition to the highly toxic direct effects of nicotine, indirect effects on blood vessels and the oxygen supply have been believed to be responsible for several adverse reactions in tendon healing. Thickness and strain ratio measurements of tendons were reduced (thinner and harder) in smokers, resulting in many clinical implications of these morphologic and elastographic changes^16^.

Equinus is the primary mechanical common denominator that leads to the majority of acquired non-traumatic foot and ankle problems by indirect leveraged means as well as direct forces along the posterior/plantar chain^17^. Reduced dorsiflexion of the ankle requires compensation within the foot, which requires subtalar joint pronation to allow the midtarsal joint to pronate and dorsiflex the foot. With fully compensated ankle equinus, the foot becomes abnormally unstable, and hypermobility results when the foot is weightbearing. Muscular imbalance of the foot causes the extensor muscles to compete against the deforming force of the tight Achilles tendon, creating hammertoe contracture that are predisposed to ulcer formation. Hypermobility can also cause abnormal movement of the metatarsal heads on the plantar soft tissues that are fixed by friction between the skin and the ground. This shearing about the metatarsal heads results in increased forefoot tissue trauma and thick callus build-up which lead to ulcerations^13^.

This study found no significant association between HbA1c ranges group and equinus contracture. The finding was counterintuitive because the group C would be expected to exhibit the highest risk of developing complications. Nevertheless, this finding was not entirely on the contrary with prior studies, e.g. by Frykberg *et al*^3^, stating an association between diabetic population and equinus development. Predominantly, diabetes is still considered as etiological factor of Achilles equinus contracture. However, chronic hyperglycemia and subsequent.

AGEs accumulation seems not to be the sole source of fibrosis of the affected tendons. As mentioned above, there are numerous pathways of soft tissue fibrosis that need to be investigated with a view to clarify the equinus pathogenesis. We found there were more women in the contracture group than in non-contracture group. According to Wenzel *et al*, women had a significant decrease in ankle joint dorsiflexion with the knee extended compared with men^18^. Vance *et al* have also reported that there were women in stenosing flexor tenosyvitis group compared with those who were not, in diabetic populations, albeit the pathomechanism remained unclear^10^. The average age in patients with contracture was 59.20 ± 6.52 years, compared with 58.61 ± 6.77 years in the non-contracture group. This is attributable to decreased length of the calf muscle-tendon unit, defined as the decrease in dorsiflexion range of motion (ROM) with the knee extended, which is associated with normal aging in both men and women. The common use of the terms “decrease flexibility” or “increased stiffness” in association with the decrease in maximal passive dorsiflexion ROM implies that shortened calf muscle-tendon unit may become stiffer with aging, even in active adults without related pathologies^19^.

To the best of our knowledge, this study is the first in published literature evaluating the influence of diabetes chronicity or glycemic control to the development of fibrosis resulting in equinus contracture of Achilles tendon.

Previous studies had mentioned the tendency of equinus among diabetic patients compared with non-diabetic populations, but lacked a comparison among diabetic patient population itself.

This study has limitations. We identified our patients based on ICD-10 coding, and thus we cannot account for patients with disease who had not presented for evaluation. The confounding factors such as daily habit, comorbidities, and duration of the disease were not analysed in details. Nonetheless, we believe that our data confirm that albeit the equinus contracture is more prevalent in diabetic patients in comparison to non-diabetic populations, HbA1c levels is not the sole determinant of Achilles contracture development. HbA1c itself can be interfered by many factors, including anemia or haemoglobin disease. Severe nephropathy can reduce insulin excretion and paradoxically lead to lower HbA1c levels as well.

Further studies should have to discover the confounding variables and the efforts to address those problems. In conjunction with previous and future research, this knowledge may provide the framework to define the treatment algorithm, to potentially reduce the incidence of diabetic foot ulceration and subsequent life-altering amputations.

## Conclusion

Based on prior studies, glycemic control may influence the pathogenesis of Achilles tendon contracture so that the treatment algorithm could be defined using patients’ HbA1c results. However, in this study there were no significant association between both variables. While there were reports on increased risk of equinus in diabetic populations, this study showed that it was not associated with the elevated HbA1c levels alone among the diabetes patients. This study emphasised that the low HbA1c levels should not be overlooked as having no risk of Achilles tendon contracture, and vice versa. Considering this study as a basis in explaining the pathogenesis of diabetic ulcer, the further studies should be able to describe the other factors or allegedly relevant determinants that may affect the Achilles tendon fibrosis in diabetic patients, with or without any correlation with hyperglycemia itself.

## References

[1] Pemayun TGD, Naibaho RM (2017). Clinical profile and outcome of diabetic foot ulcer, a view from tertiary care hospital in Semarang, Indonesia.. Diabet Foot Ankle..

[2] Batista F, Nery C, Pinzur M, Monteiro AC, de Souza EF, Felippe FH (2008). Achilles tendinopathy in diabetes mellitus.. Foot Ankle Int..

[3] Frykberg RG, Bowen J, Hall J, Tallis A, Tierney E, Freeman D (2012). Prevalence of equinus in diabetic versus nondiabetic patients.. J Am Podiatr Med Assoc..

[4] Ramanujam CL, Zgonis T (2017). Surgical Correction of the Achilles Tendon for Diabetic Foot Ulcerations and Charcot Neuroarthropathy.. Clin Podiatr Med Surg..

[5] Primadhi RA (2021). Susceptibility Factors for Early Reamputation in Diabetic Great Toe Gangrene.. Curr Diabetes Rev..

[6] Ban CR, Twigg SM (2008). Fibrosis in diabetes complications: pathogenic mechanisms and circulating and urinary markers.. Vasc Health Risk Manag..

[7] Canguven O, Talib R, El Ansari W, Khalafalla K, Al Ansari A (2018). Is Hba1c level of diabetic patients associated with penile prosthesis implantation infections?. Aging Male..

[8] Diabetes Control, Complications Trial Research Group, Nathan DM, Genuth S, Lachin J, Cleary P, Crofford O (1993). The effect of intensive treatment of diabetes on the development and progression of long-term complications in insulin-dependent diabetes mellitus.. N Engl J Med..

[9] Choudhury M, Taylor P, Morgan PH, Duckers J, Lau D, George L (2019). Association between HbA1c and the development of cystic fibrosis-related diabetes.. Diabet Med..

[10] Vance MC, Tucker JJ, Harness NG (2012). The association of hemoglobin A1c with the prevalence of stenosing flexor tenosynovitis.. J Hand Surg Am..

[11] Brenner DA (2013). Reversibility of liver fibrosis.. Gastroenterol Hepatol (N Y)..

[12] Gatt A, Chockalingam N (2012). Assessment of Ankle Joint Dorsiflexion: An overview.. Rev Int Cienc Podol..

[13] Van Gils CC, Roeder B (2002). The effect of ankle equinus upon the diabetic foot.. Clin Podiatr Med Surg..

[14] Florkowski C (2013). HbA1c as a Diagnostic Test for Diabetes Mellitus- Reviewing the Evidence.. Clin Biochem Rev..

[15] Goldin A, Beckman JA, Schmidt AM, Creager MA (2006). Advanced glycation end products: sparking the development of diabetic vascular injury.. Circulation..

[16] Agladıoglu K, Akkaya N, Gungor HR, Akkaya S, Ok N, Ozcakar L (2016). Effects of Cigarette Smoking on Elastographic Strain Ratio Measurements of Patellar and Achilles Tendons.. J Ultrasound Med..

[17] Amis J (2016). The Split Second Effect: The Mechanism of How Equinus Can Damage the Human Foot and Ankle.. Front Surg..

[18] Wenzel EM, Kajgana Z, Kelley KD, Mason KM, Wrobel JS, Armstrong DG (2009). Prevalence of Equinus in Patients Diagnosed with Plantar Fasciitis.. FAOJ..

[19] Gajdosik RL, Vander Linden DW, Williams AK (1999). Influence of age on length and passive elastic stiffness characteristics of the calf muscle-tendon unit of women.. Phys Ther..

